# Retinitis Pigmentosa and Retinal Neovascularization in a Patient With a Heterozygous Mutation in the CRB1 Gene: A Case Report

**DOI:** 10.7759/cureus.82814

**Published:** 2025-04-22

**Authors:** Joel Castro, Andres Emanuelli, Natalio Izquierdo

**Affiliations:** 1 Department of Ophthalmology, Emanuelli Research ＆ Development, Arecibo, PRI; 2 Department of Ophthalmology, Retina Care, San Juan, PRI; 3 Department of Microbiology, Universidad de Puerto Rico en Arecibo, Arecibo, PRI; 4 Department of Ophthalmology, University of Puerto Rico, Medical Sciences Campus, San Juan, PRI; 5 Department of Surgery, University of Puerto Rico, Medical Sciences Campus, San Juan, PRI

**Keywords:** compound heterozygote, crb1, hereditary retinal diseases, retinal neovascularization, retinitis pigmentosa

## Abstract

This report describes the cases of two siblings who both experienced reduced visual acuity in both eyes since adolescence, along with night blindness and progressive peripheral vision loss. Fundus photography revealed a “salt-and-pepper” appearance around the macula, consistent with rod-cone dystrophy, while fluorescein angiography showed neovascularization of the optic disc and mid-peripheral retina. Optical coherence tomography showed parafoveal macular thickening, mild intraretinal fluid, and loss of the inner segment/outer segment layer. Genetic testing identified a compound heterozygous mutation in the *CRB1* gene in both patients. This case underscores the phenotypic variations in patients with mutations in *CRB1*. To our knowledge, this is the first report of optic disc neovascularization in CRB1 compound heterozygotes. Further phenotypic and genotypic evaluations are necessary to assess ocular complications in patients with retinitis pigmentosa, including those involving retinal pigment epithelium atrophy.

## Introduction

Retinitis pigmentosa (RP) is a group of inherited retinal diseases that lead to blindness. Patients usually have a loss of night vision, a gradual loss of peripheral vision, central vision, and color vision. Patients with RP benefit from a comprehensive ophthalmic evaluation, electroretinography, visual field testing, and optical coherence tomography (OCT) [1]. 

There are two types of ophthalmic new vessel growth (neovascularization): retinal neovascularization and choroidal (or subretinal) neovascularization (NV). The former occurs when retinal ischemia is secondary to retinal vessel disease. The latter occurs in diseases of the outer retinal and Bruch's membrane [2]. 

The* *Crumbs homolog 1 *(CRB1) *gene is critical for the normal development of the photoreceptors. The CRB1 protein helps determine the structure, orientation, and connections of photoreceptors with other retinal cells. Patients with mutations in the *CRB1 may* have various phenotypes, including Leber congenital amaurosis (LCA), to rod-cone dystrophies. Retinal dystrophies associated with *CRB1* mutations may have preservation of the para-arteriolar retinal pigment epithelium (PPRPE) and retinal telangiectasia with exudation (also referred to as Coats-like vasculopathy) [3]. 

Retinal dystrophies associated with *CRB1* mutations are inherited as an autosomal recessive trait. Patients who are homozygous or compound heterozygous for mutations in this gene may have affected phenotypes. So far, more than 200 mutations in this gene have been reported [4,5]. 

The prevalence of *CRB1-*associated diseases is approximately one in 86,500 in the United States. It has been reported that the prevalence increases to one in 3,000 worldwide. Mutations in the *CRB1* gene lead to approximately 10% of patients with LCA and up to 6.5% of patients with RP [6]. 

RP, Joubert syndrome, and Zellweger syndrome are all of the differential diagnoses of LCA. Interestingly, this mutation in the *CRB1* gene may lead to two of the abovementioned phenotypes. For this reason, phenotypic and genotypic studies are needed in these patients [7].

We report on two siblings with compound heterozygous *CRB1* mutations who developed bilateral neovascularization, along with preretinal and intraretinal hemorrhages in both eyes. 

## Case presentation

Patient 1

The patient was a 24-year-old man with progressively worsening visual symptoms due to floaters. He experienced reduced visual acuity in both eyes since the age of 15, along with night blindness and a painless, gradual loss of peripheral vision. His condition had progressed to the point where he could no longer play sports or drive his car.  He underwent a comprehensive ophthalmic evaluation. His best-corrected visual acuity (BCVA) was 20/200 in both eyes. Refraction measured -2.00 -1.25x 125˚ in the right eye and -1.25 -0.75 x 30˚ in the left eye. 

**Figure 1:**
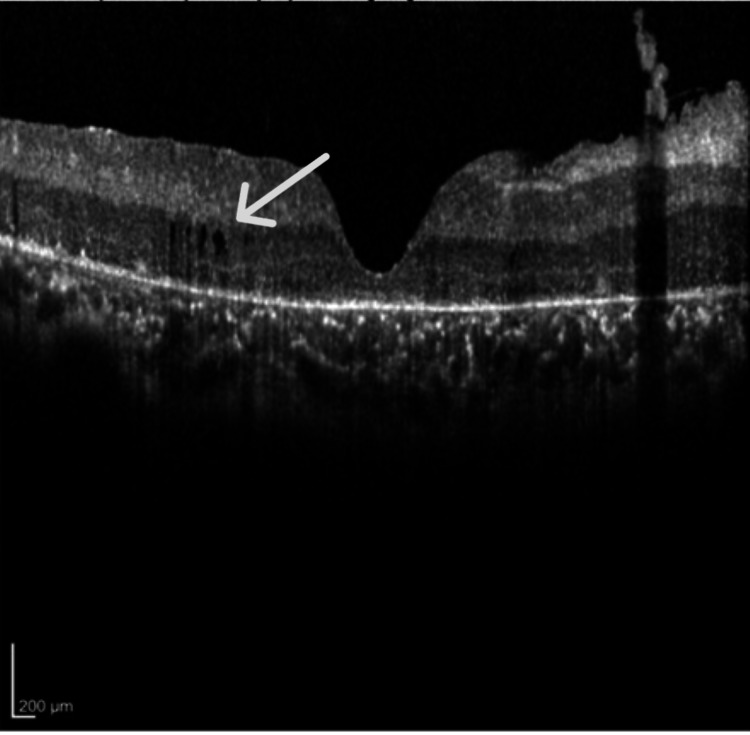
Optical coherence tomography of the right eye shows parafoveal macular thickening with mild intraretinal fluid and loss of inner segment/outer segment, consistent with an advance stage of retinitis pigmentosa.

**Figure 2:**
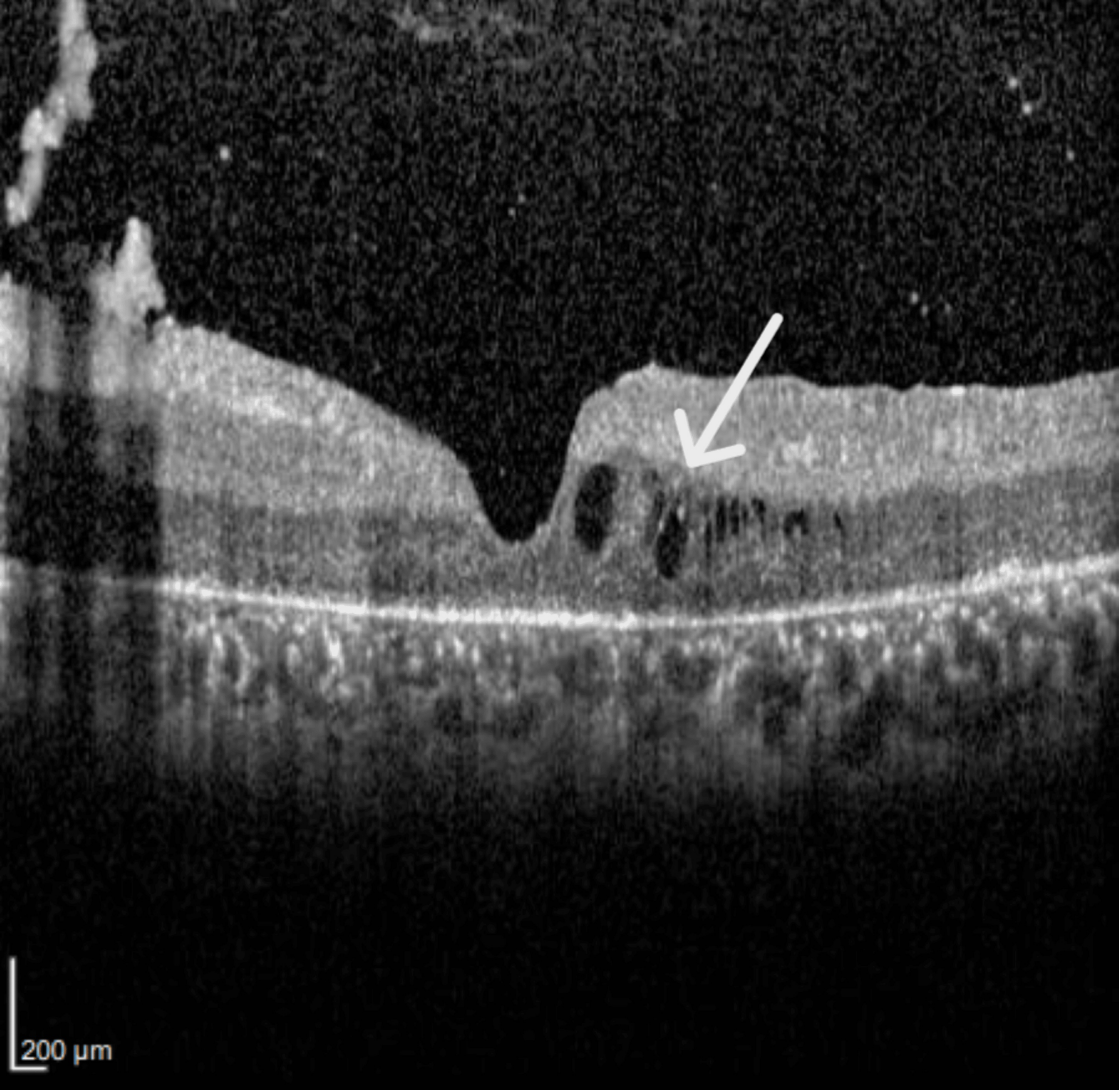
Optical coherence tomography of the left eye shows parafoveal macular thickening with mild intraretinal fluid and loss of inner segment/outer segment, consistent with an advance stage of retinitis pigmentosa.

OCT (Spectralis OCT; Heidelberg Engineering, Inc., Heidelberg, Germany) was done, revealing parafoveal macular thickening with mild intraretinal fluid and loss of inner segment/outer segment (IS/OS) layer (Figures 1, 2). Macular thickness measured 285 microns in the right eye and 290 microns in the left eye. The total macular volume measured 11.94 mm³ in the right eye and 11.93 mm³ in the left. The mean deviation was -27.33dB p < 0.5% in the right eye and -13.51dB p < 0.5% in the left eye.

**Figure 3:**
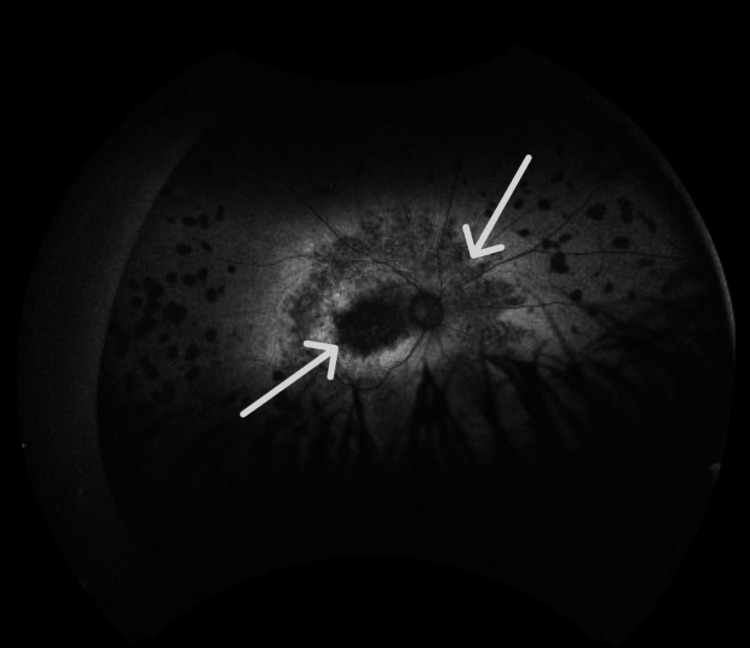
Fundus autofluorescence of right eye shows parafoveal macular thickening with mild intraretinal fluid and loss of inner segment/outer segment, consistent with an advance stage of retinitis pigmentosa.

**Figure 4:**
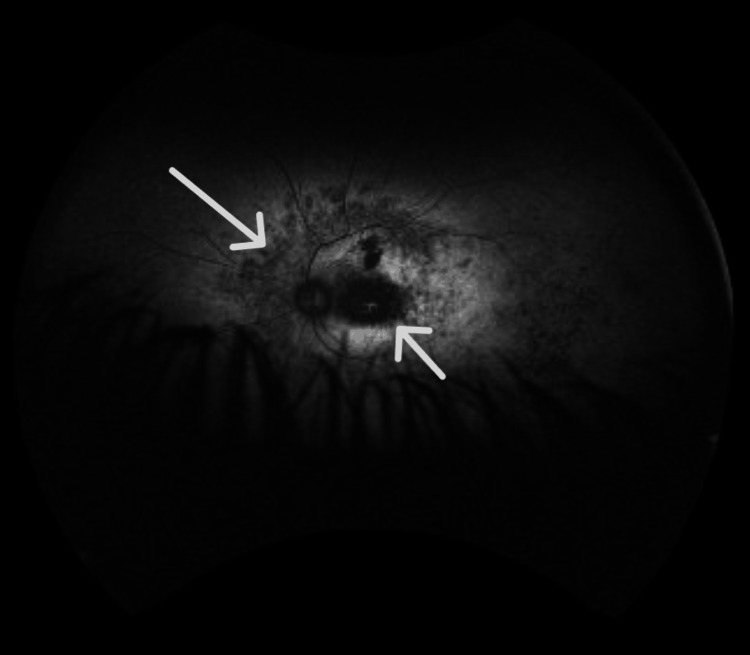
Fundus autofluorescence of the left eye shows regions of mottled hypo-autofluorescence that also include the fovea, consistent with loss of the photoreceptors due to progression of retinitis pigmentosa.

Autofluorescence imaging (Figures 3, 4) revealed regions of normal or increased autofluorescence, along with areas of mottled hypo-autofluorescence that include the fovea. 

**Figure 5:**
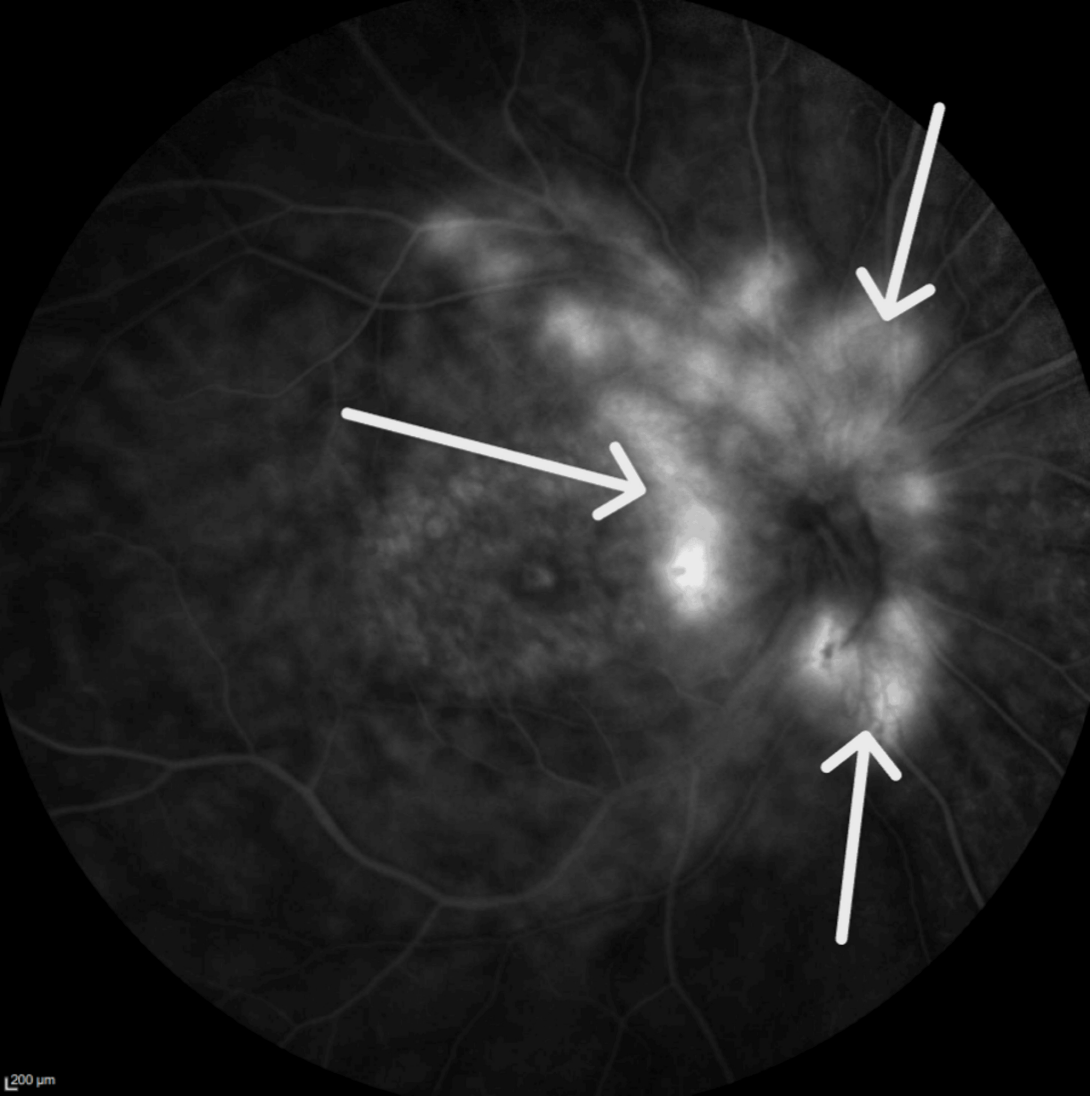
Fluorescein angiography of right eye shows several areas of hyperflouresence along the optic nerve and the arcade vessels, consistent with neovascular vessels.

**Figure 6:**
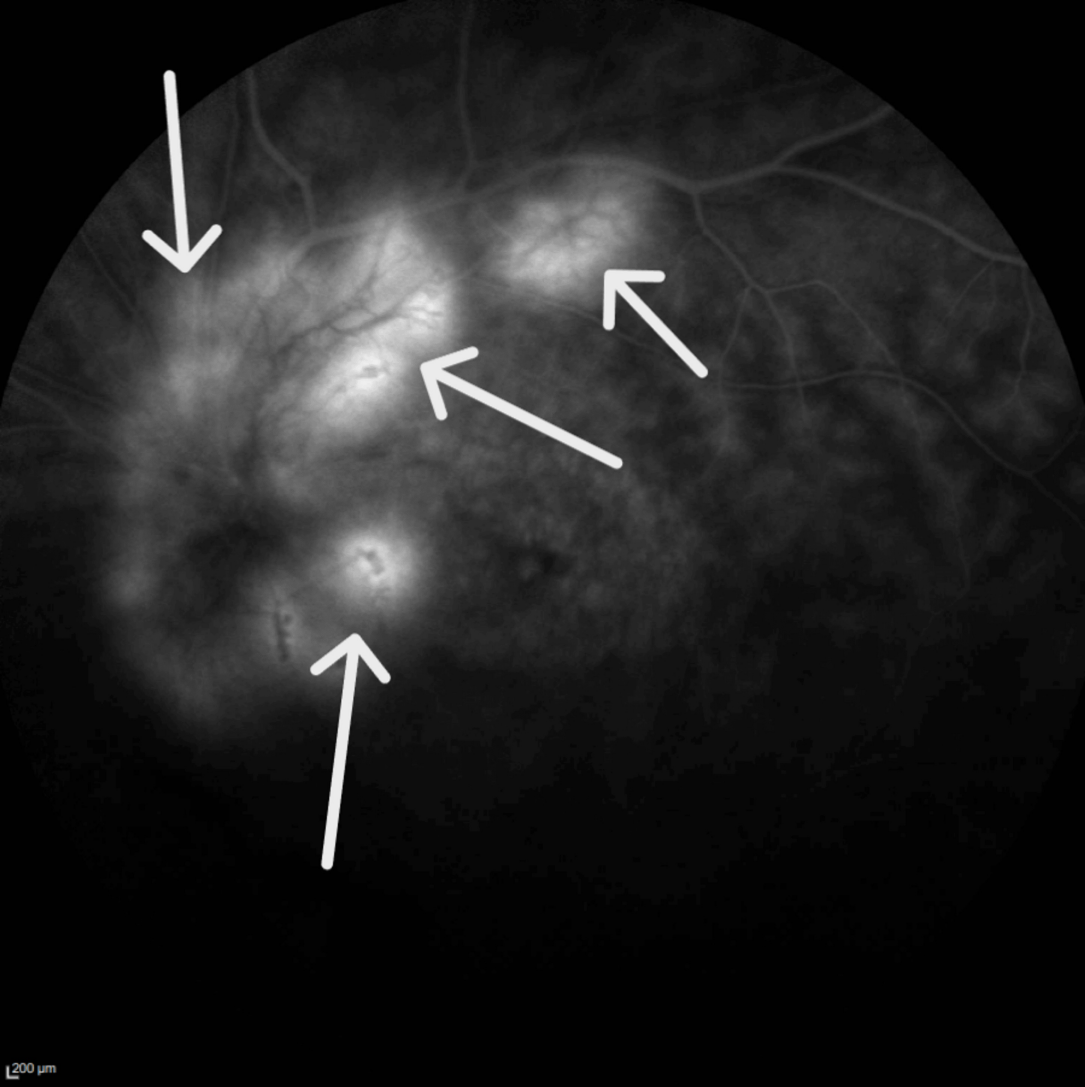
Fluorescein angiography of the left eye shows several areas of hyperflouresence along the optic nerve and the arcade vessels, consistent with neovascular vessels.

Fundus angiography revealed multiple areas of hyperfluorescence along the optic nerve and arcade vessels, with diffuse capillary dropout (Figures 5, 6). Additionally, diffuse late leakage occurred in the macular area. 

A clinical diagnosis of RP was made, and a saliva sample was submitted for genetic testing, including gene sequencing and deletion/duplication analysis using next-generation sequencing (NGS) by Invitae Corp., San Francisco, California, United States. The results showed that the patient was a compound heterozygote with mutations in the *CRB1* gene, specifically the variants: c.481del, p.(Ala161Profs*45), and c.498_506del, p.(Ile167_Gly169del). 

Patient 2

A 23-year-old female patient, the sister of Patient 1, presented with similar symptoms of nyctalopia and peripheral vision loss. There was no history of consanguinity. Both parents are deceased. The patient underwent a comprehensive ophthalmic evaluation, revealing a best-corrected visual acuity of 20/200 in both eyes. Refraction measurements were as follows: right eye -0.75 -1.50x 10˚ and left eye +0.50 -1.50 x 7˚. 

**Figure 7:**
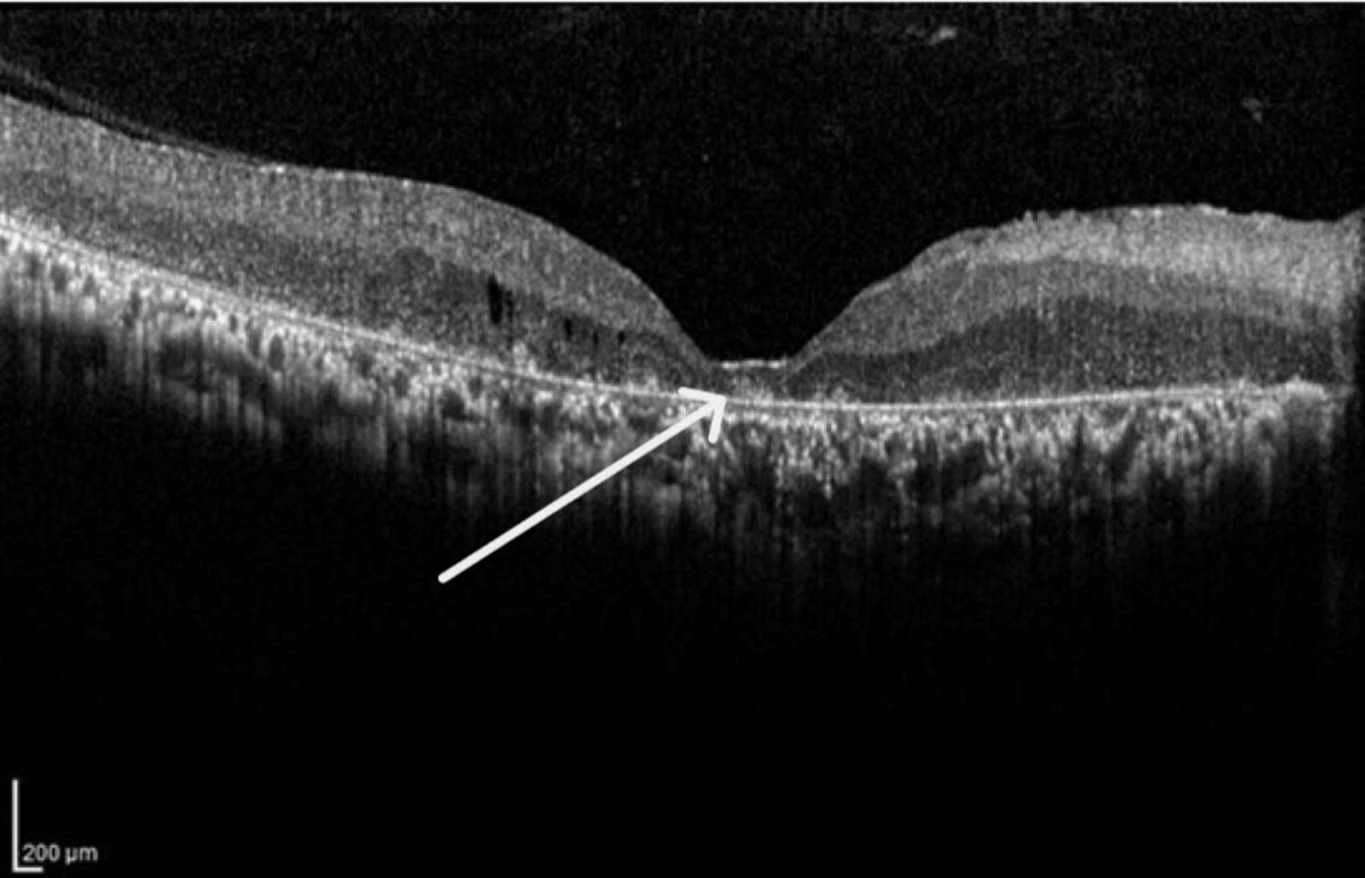
Optical coherence tomography of the right eye shows mild intraretinal fluid and loss of inner segment/outer segment, consistent with an advance stage of retinitis pigmentosa.

**Figure 8:**
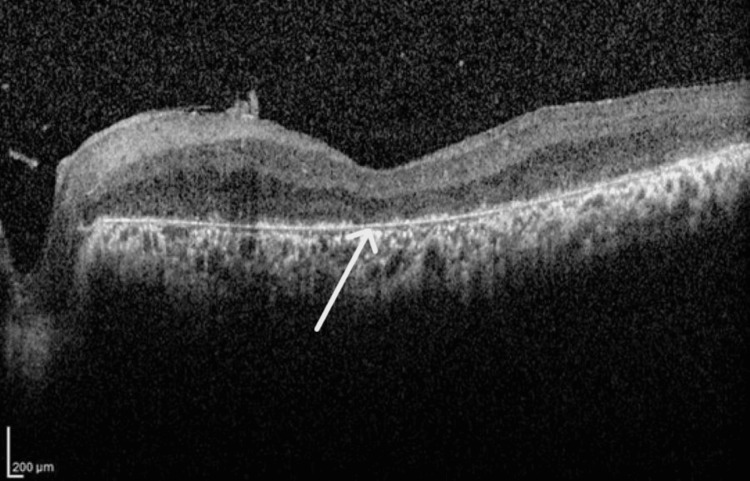
Optical coherence tomography of the left eye shows mild intraretinal fluid and loss of inner segment/outer segment, consistent with an advance stage of retinitis pigmentosa.

OCT (Spectralis OCT) images showed parafoveal macular thickening with mild intraretinal fluid and loss of IS/OS layer (Figures 7, 8). The macular thickness was measured at 208 microns in the right eye and 212 microns in the left eye. The total macular volume measured 9.54 mm³ in the right eye and 9.55. mm³ in the left eye. The mean deviation was -2.99dB p < 2% in the right eye and -2.92dB p < 2% in the left eye.

**Figure 9:**
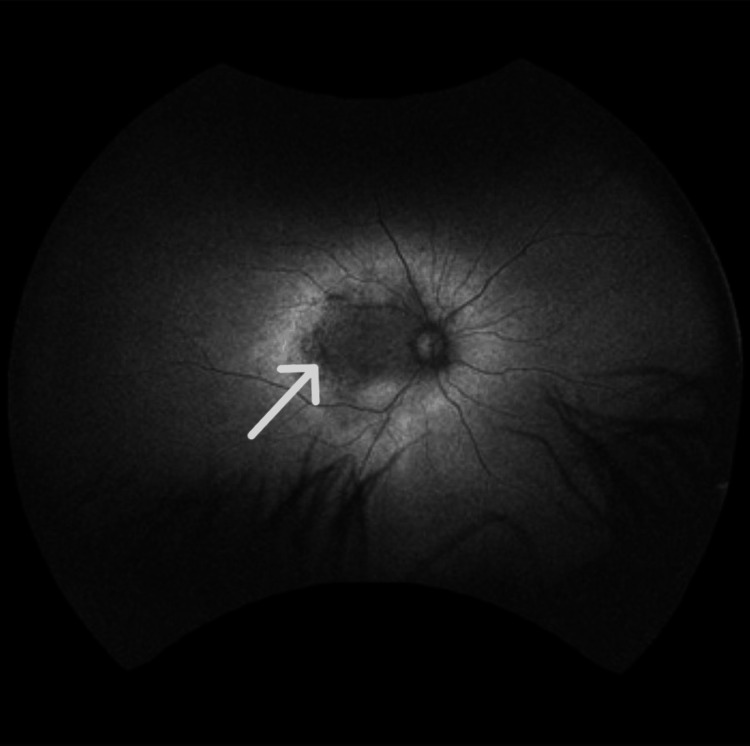
Fundus autofluorescence of the right eye shows regions of mottled hypo-autofluorescence that also include the fovea, consistent with loss of the photoreceptors due to progression of retinitis pigmentosa.

**Figure 10:**
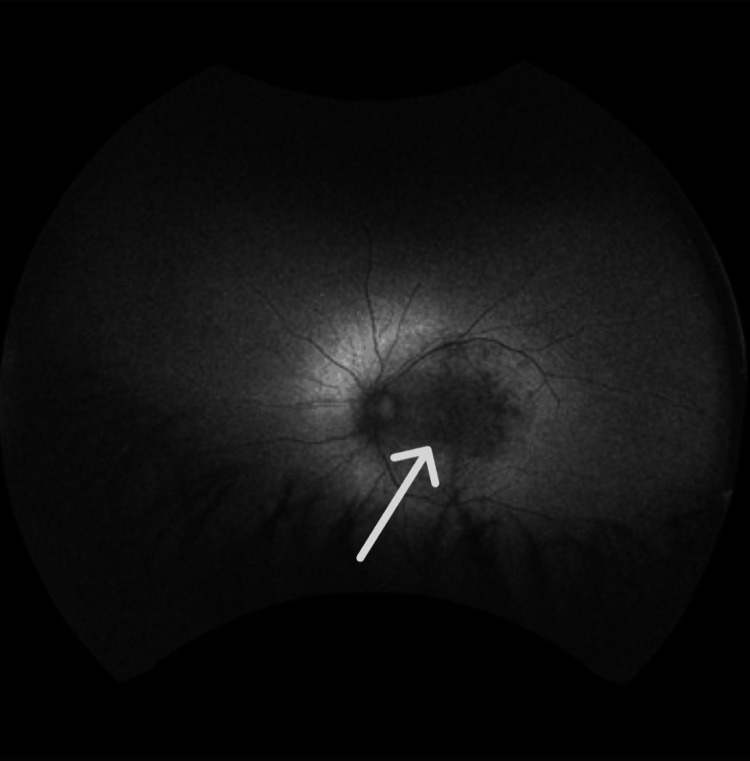
Fundus autofluorescence of the left eye shows regions of mottled hypo-autofluorescence that also include the fovea, consistent with loss of the photoreceptors due to progression of retinitis pigmentosa.

Autofluorescence imaging showed areas of normal or increased autofluorescence, along with regions of mottled hypo-autofluorescence, including the fovea (Figures 9, 10).

**Figure 11:**
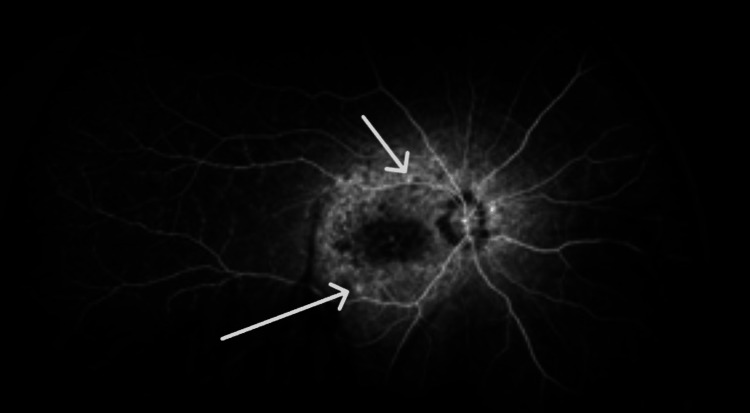
Fluorescein angiography in the right eye shows small areas of hyperflouresence along the arcade vessels, consistent with neovascular vessels.

**Figure 12:**
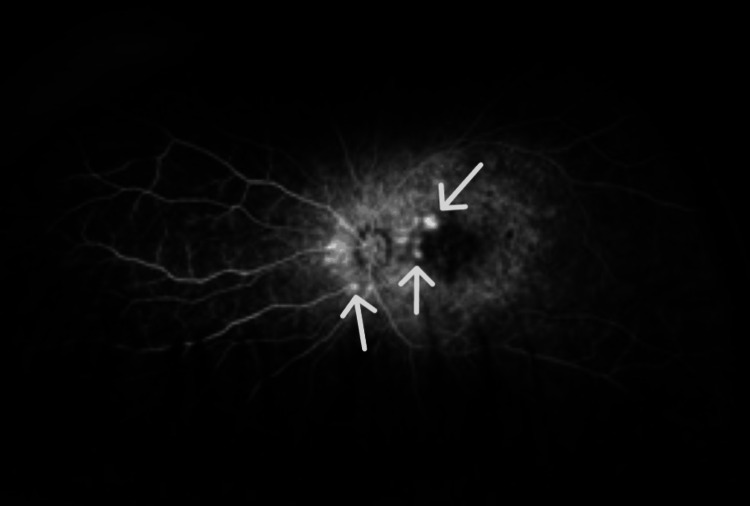
Fluorescein angiography in the left eye shows small areas of hyperflouresence along the nasal parafoveal area, consistent with neovascular vessels.

Fundus angiography showed several areas of hyperfluorescence along the optic nerve and arcade vessels, accompanied by diffuse capillary dropout (Figures 11, 12). Additionally, diffuse late leakage was observed in the macular region. 

Since a clinical diagnosis of RP was reached, a saliva sample was submitted for genetic testing, including gene sequencing and deletion/duplication analysis using NGS by Invitae Corp. showed that the patient was a compound heterozygote with mutations in the *CRB1* gene with variants: c.481del, p.(Ala161Profs*45), and c.498_506del, p.(Ile167_Gly169del).

## Discussion

Previous studies have reported that visual acuity and visual field loss occur in patients with RP [8]. In the siblings in this case report, best-corrected visual acuities were reduced, and visual fields' mean deviations were statistically significantly decreased. These findings are compatible with the end stage of the disease. 

Nguyen et al. reported that patients with RP exhibit macular OCT findings, including cystoid macular edema (CME) and loss of the IS/OS layers [9]. OCT imaging of the patients in the current report showed several significant findings. First, parafoveal macular thickening was observed, indicating localized swelling in the central retina. Additionally, mild intraretinal fluid was detected, suggesting fluid accumulation within the retinal layers. Most notably, there was a discernible loss of the IS/OS layer, which is critical for photoreceptor function. These findings are consistent with existing scientific literature. 

Previous studies have documented that patients with mutations in the *CRB1* gene may have phenotypes including LCA, rod-cone dystrophy, and PPRPE [10]. Our patients were diagnosed with RP. Genetic testing revealed that both patients were compound heterozygotes with mutations in the *CRB1* gene, specifically the variants: c.481del, p.(Ala161Profs*45), and c.498_506del, p.(Ile167_Gly169del). To our knowledge, the former mutation has not been reported in the ClinVar database, whereas the latter has been described [11]. 

A noteworthy association has been reported between RP and two distinct types of neovascularization: sea fan-type retinal neovascularization [12] and choroidal neovascularization [13]. The siblings in the current report had both optic disk and peripheral neovascularization in the arcade vessels of both eyes. This combination is notably distinct when compared to existing literature on similar conditions. Further investigation and analysis are needed to understand the underlying mechanisms and clinical implications of this novel presentation. Understanding these vascular changes is crucial for managing patients with RP and developing targeted interventions to preserve vision. 

Figures 5, 6 demonstrate that Patient 1 had neovascularization in the disc area. To our knowledge, this is the first report of neovascularization at the disc in a patient with RP.  Figures 11, 12 show that Patient 2 had small areas of hyperfluorescence along the arcade vessels in the right eye, as well as areas of hyperfluorescence in the nasal parafoveal area of the left eye, consistent with neovascularization. These findings are similar to those observed in Patient 1.  

The findings of the patients in this report are consistent with existing literature regarding macular findings and the association of *CRB1* mutations with RP [14]; however, the absence of other typical *CRB1*-associated phenotypes highlights the condition's phenotypic variability. Optic disk and retinal neovascularization have not been previously described, suggesting a novel presentation. This phenomenon may be attributed to retinal ischemia secondary to photoreceptor degeneration and retinal pigment epithelium atrophy, potentially exacerbated by *CRB1*-induced disruptions in the blood-retinal barrier [15]. 

These two cases underscore the importance of comprehensive retinal examinations in patients with RP, particularly those with *CRB1* mutations, to facilitate early detection and management of potential complications due to neovascularization. Given the role of vascular endothelial growth factor (VEGF) in neovascularization, treatment with anti-VEGF agents may be a viable option for managing the retinal neovascularization in patients with these complications [16]. Further research is warranted to investigate the prevalence, natural history, and underlying mechanisms of retinal neovascularization in RP, as well as to evaluate potential treatment strategies, including the use of anti-VEGF therapy.

## Conclusions

Understanding the various phenotypic presentations in patients with *CRB1* gene mutations remains a significant challenge for researchers and healthcare providers. The complex relationship between clinical manifestations and genetic variations requires thorough investigation. Patient 1 had optic disk neovascularization, and Patient 2 had peripheral retinal neovascularization. To our knowledge, this is the first report of this complication in patients with compound heterozygous *CRB1* mutations. These highlight the diverse manifestations associated with *CRB1 *mutations. These findings underscore the need for further exploration into the mechanisms behind the phenotypic diversity in patients with *the CRB1* gene related to retinitis pigmentosa. Comprehensive retina evaluation, including fluorescein angiography, is essential for accurate diagnosis and effective management in such patients. 
